# Co-occurrence and clustering of the four major non-communicable disease risk factors in Brazilian adolescents: Analysis of a national school-based survey

**DOI:** 10.1371/journal.pone.0219370

**Published:** 2019-07-03

**Authors:** Camila Zancheta Ricardo, Catarina Machado Azeredo, Leandro Fórnias Machado de Rezende, Renata Bertazzi Levy

**Affiliations:** 1 Departamento de Medicina Preventiva, Faculdade de Medicina FMUSP, Universidade de São Paulo, São Paulo, SP, Brazil; 2 Faculdade de Medicina, Universidade Federal de Uberlândia, Uberlândia, MG, Brazil; East Tennessee State University, UNITED STATES

## Abstract

**Background:**

The major non-communicable chronic diseases (NCD) are associated with a small group of modifiable lifestyle-related risk factors, including smoking, insufficient physical activity, unhealthy eating, and alcohol abuse. In this study, we evaluated the co-occurrence and clustering of the major NCD risk factors among Brazilian adolescents.

**Methods:**

This cross-sectional study analyzed data of 101,607 adolescents from the Brazilian National Survey of School Health (PeNSE) 2015. The risk factors included were: regular consumption of ultra-processed foods, irregular consumption of fruits and vegetables, insufficient physical activity, smoking, and alcohol consumption. Clustering was defined through the ratio between observed and expected prevalences of combination of risk factors greater than 1. Expected prevalence of the co-occurrence of risk factors was calculated from the joint probability of the behaviors. Additionally, we examined the presence of at least four risk factors according to socioeconomic characteristics.

**Results:**

Of the 32 combinations of risk factors, 13 corresponded to clustering. We observed a strong correlation between alcohol consumption and smoking, which were found together in 8 of the 13 clusters identified. The most frequent combinations of risk factors involved unhealthy eating and insufficient physical activity. Only 2.9% of the adolescents did not present any risk behaviors, while 38.0%, 32.9%, 9.4% and 1.8% accumulated two, three, four and five risk factors, respectively. The accumulation of risk factors was higher in girls, older adolescents, those who did not live with both parents, children of less-educated mothers, students attending public school, and residents of cities in more developed urban areas of the country.

**Conclusions:**

The main risk factors for NCD are frequent and not randomly distributed among Brazilian adolescents. Our results provide information for policymakers to target specific groups and joint behavioral risk factors for health improvement in adolescents.

## Introduction

Non-communicable diseases (NCD) are the leading cause of death worldwide and impact both quality of life and social and economic development, particularly in low and middle-income countries [1]. A recent analysis of the burden of disease showed that NCD have grown significantly in Brazil between 1990 and 2016, and have become the leading cause of death and years of life lost [2]. Similarly, the contribution of NCD risk factors to disability-adjusted life years (DALY) sharply increased in this period. In 2016, the main risk factors that contributed to DALY in Brazil were alcohol and drug use, high blood pressure, high body mass index, inadequate diet, smoking and low physical activity [2].

These main risk behaviors are often acquired during adolescence and tend to remain in adulthood [3, 4]. In addition, epidemiological studies suggest an association between risk factors during adolescence and the development of NCD later in life, regardless of exposures in adulthood [5–7]. Therefore, it is important to monitor NCD risk factors in adolescents, including their co-occurrence in the population, as risk factors can interact with each other, thereby producing greater risk than the sum of individual risks [8–11].

Despite the increasing number of studies aimed at identifying how major NCD risk and protective behaviors are related, the majority of the studies focus mainly on the adult population [12–16] with some studies focusing on adolescents in developed countries [17–20]. The literature in this field is highly heterogeneous, with different methodologies and risk factors assessment and definition, and no consensus about which risk factors usually occur together [21, 22].

In Brazil, the co-existence of NCD risk factors among adolescents has been studied previously [23–27]. For example, clustering of risk factors including physical activity, sedentary behavior, and diet has been reported using data from a school-based national and representative survey [25, 26]. Another study evaluated patterns of multiple health-related behaviors including diet, physical activity, alcohol consumption, smoking, drug use, aggressive behavior, and unsafe sex, exploring the correlation between these behaviors [27]. These studies provided some evidence on how risk factors interact in Brazilian adolescents, but they did not provide information regarding the prevalence and co-occurrence of all the four main risk factors for NCD.

In this study, the two primary objectives were: 1) to evaluate the prevalence and clustering of NCD risk factors (smoking, insufficient physical activity, unhealthy eating, and alcohol abuse); and 2) to verify the co-occurrence of risk factors according to the sociodemographic characteristics of Brazilian adolescents.

## Methods

### Design, study population, and data collection

We used data from the Brazilian National Survey of School Health (*Pesquisa Nacional de Saúde do Escolar*—PeNSE) 2015, a cross-sectional and representative survey of ninth grade students from public and private schools in Brazil [28]. PeNSE enrolled a total of 102,072 students from 3,040 schools all over the country. The sample weight of each student was calculated to represent all students who attended classes regularly [29]. PeNSE collected data through a structured questionnaire (self-administered), based on adaptations of the Global School-Based Student Health Survey and the Youth Risk Behavior Surveillance System to the reality of Brazil. Details of the sampling procedure, as well as the complete questionnaire, are available elsewhere [28]. In this study, we used data from 101,607 students with complete information on five NCD risk factors analyzed.

PeNSE was approved by the National Commission of Research Ethics (Comissão Nacional de Ética em Pesquisa–Conep), record no. 1.006.467. The survey was performed in accordance with the Declaration of Helsinki and all participants gave their informed consent. The database was made publicly available on a Brazilian Institute of Geography and Statistics website without any information that could identify subjects.

### Description of variables

The five risk factors used in this study were defined as presented in Table 1. The World Health Organization (WHO) recommends 60 minutes a day of moderate to vigorous physical activity for adolescents [30]. We adopted less than 300 minutes weekly as the cutoff point for physical inactivity, which was previously validated [31]. Adolescents are more vulnerable than adults to harms caused by tobacco and alcohol consumption [32, 33]. For this reason, the sale of tobacco and alcohol are prohibited for this age group in Brazil. We considered any consumption of tobacco and alcohol in the previous month a risk factor for NCD in adolescents. For dietary risk factors, we have used the concept of ‘regular consumption’ (≥5 times in the past week), which was validated using 24-hour recall among adolescents [34]. Low consumption of fruits and vegetables is strongly recognized by WHO as a risk factor for NCD [35], therefore, we used the complementary idea of irregular consumption (<5 times in the past week) as the risk factor [36]. On the other hand, the consumption of ultra-processed food has been recently suggested to be related to low quality diet, obesity, and NCD [37–39]. Furthermore, the Brazilian Dietary Guideline recommends avoiding the consumption of ultra-processed foods [40]. For this reason, we choose to consider consumption of ultra-processed foods ≥ 5 days a week as a risk factor.

**Table 1:** Indicator of risk factors used in the present study.

Risk factor	Assessment in the survey	Definition applied
Insufficient physical activity	Physical activity was estimated by multiplying the mean of time spent walking to school, leisure physical activities and scholar physical activities by the weekly frequency in the last 7 days.	Physical activity for less than 300 minutes per week.
Smoking	Question about the frequency of smoking (number of days) in the previous month	Smoking one or more days in the last month.
Alcohol consumption	Question about the frequency (number of days) of consumption of at least one alcoholic drink in the last 30 days.	Alcohol consumption one or more days in the month.
Regular consumption of ultra-processed foods	Questions regarding the frequency of consumption (number of days) of the following ultra-processed food consumption in the last 7 days: sweets, soft drinks and salty ultra-processed foods (hamburger, ham, mortadella, salami, sausage, hot dog sausage, instant noodles, salty crackers).	Consumption of any of the listed ultra-processed items in five or more days in the week.
Irregular consumption of fruits and vegetables	Questions regarding the frequency consumption (number of days) in the last 7 days of fruits and vegetables.	Consumption of fruits or vegetables in four or fewer days in the week.

The socioeconomic and demographic variables used in this study were: sex, age (<14 years, 14 to 16 years or ≥16 years), skin color (white or non-white), living arrangement (living with both parents, only one of parent, or with neither parent), maternal education (no formal education, incomplete primary education, complete primary education, high school or college), type of school (public or private), type of municipality (capital or non-capital), area of municipality (urban or rural), region of the country (less developed—North, Northeast, or more developed—South, Southeast, Midwest), and goods and services score.

To calculate goods and services score, the following items were considered: landline phone, cell phone, computer, automobile, internet service, access to household toilet and maid service three or more days per week. Each item received a weight equivalent to the inverse of its prevalence in the sample. The score of the adolescents was the sum of the weights of their accessible items [41]. For analysis, the goods and services score was divided into terciles.

### Statistical analysis

For the statistical analysis in this study, risk factors were coded as binary variables (presence or absence). The prevalence of co-occurrence of risk factors was calculated using the joint probability of the behaviors presented. The presence of clustering was studied using a comparison between observed (O) and expected (E) prevalences. The expected prevalence for each combination was calculated by multiplying the probabilities of each defined risk factor, based on its distribution in the studied population.

Thirty-two possible combinations of the five risk factors were studied. Clustering was defined when a combination was more prevalent than expected, based on the prevalence of each isolated risk, i.e. a combination in which the ratio O/E was greater than 1 [22]. Confidence intervals (CI) for O/E ratios were obtained by Newton's method assuming Poisson distribution [42], and we considered clusters those combinations in which 95% CI did not contain the null value.

The variable maternal education, which originally had 27% missing data, was submitted to multiple imputation by chained equations. Socioeconomic and risk variables served as predictors in the imputation, because they would be part of subsequent analysis, as recommended in the literature [43]. The imputed data presented satisfactory statistical reproducibility according to the Monte Carlo error analysis [44].

Each of the risk factors and the accumulation of at least four of them were described according to socioeconomic characteristics. The analyses were conducted using the Stata software version 14.1 and Microsoft Excel and took the sample design into consideration.

## Results

The most frequent risk factor among the students was irregular consumption of fruits and vegetables (80.7%), followed by insufficient physical activity (65.6%), regular consumption of ultra-processed foods (60.6%), alcohol consumption (23.8%) and smoking (5.6%) (Table 2).

**Table 2:** Prevalence of single risk factors in Brazilian adolescents, by socioeconomic variables. Brazilian National Survey of School Health (PeNSE), 2015.

Variables	Prevalence of risk factors														
	Insufficient physical activity			Smoking			Alcohol consumption			Irregular consumption of fruits and vegetables			Regular consumption of ultra-processed foods		
	%	95% CI		%	95% CI		%	95% CI		%	95% CI		%	95% CI	
Sex															
Boys	56.1	55.1	57.0	5.8	5.3	6.3	22.4	21.7	23.2	80.6	79.9	81.2	57.6	56.8	58.4
Girls	74.6	73.7	75.5	5.3	4.9	5.8	25.1	24.3	25.9	80.8	80.1	81.5	63.4	62.5	64.3
Age															
13 years or less	67.7	73.7	75.5	2.7	2.2	3.4	16.3	15.0	17.7	79.9	78.5	81.2	62.8	61.3	64.3
14–15 years	65.0	64.2	65.8	5.4	5.0	5.8	24.0	23.3	24.7	80.5	78.5	81.2	60.7	60.0	61.4
16 years or more	65.8	64.3	67.2	11.7	10.6	12.8	35.0	33.5	36.5	83.5	82.3	84.6	56.3	54.7	57.8
*p* trend	0.036			<0.001			<0.001			0.001			<0.001		
Skin color															
White	66.1	65.0	67.1	5.1	4.7	5.6	23.9	23.0	24.9	79.9	78.9	80.8	61.5	60.4	62.6
Non-white	65.3	64.4	66.1	5.8	5.4	6.2	23.7	23.0	24.4	81.2	80.6	81.7	60.1	59.3	60.8
Living with parents															
Both parents	66.3	65.4	67.2	4.4	4.0	4.8	21.1	20.3	21.8	79.9	79.2	80.6	59.7	58.8	60.5
One parent	64.1	63.0	65.1	7.0	6.4	7.5	27.3	26.4	28.3	81.8	81.0	82.5	62.1	61.2	63.0
Neither parent	66.8	64.6	68.8	9.0	7.9	10.4	30.5	28.7	32.3	82.2	80.5	83.7	60.8	58.8	62.7
*p* trend	0.032			<0.001			<0.001			<0.001			0.001		
Goods and service score															
1 tercile	68.1	67.2	69.0	5.7	5.3	6.2	21.7	20.9	22.4	84.0	83.4	84.7	54.7	53.8	55.5
2 tercile	65.7	64.6	66.8	5.4	5.3	6.2	24.2	23.2	25.1	80.5	79.6	81.3	62.6	61.6	63.6
3 tercile	62.2	60.9	63.5	5.6	5.0	6.2	26.1	24.9	27.2	76.8	75.7	77.8	66.0	64.9	67.1
*p* trend	<0.001			0.612			<0.001			<0.001			<0.001		
Type of school															
Private	65.6	64.1	67.1	3.6	3.0	4.3	21.2	19.6	23.0	78.5	76.8	80.1	65.6	64.0	67.1
Public	65.6	64.7	66.4	5.9	5.5	6.3	24.2	23.6	24.9	81.1	80.5	81.6	59.7	59.0	60.4
Area of municipality															
Rural	75.4	73.4	77.3	4.2	3.5	5.0	20.8	19.1	22.6	83.1	81.7	84.4	48.8	46.6	51.0
Urban	64.7	63.9	65.5	5.7	5.3	6.1	24.1	23.4	24.7	80.5	79.9	81.0	61.7	61.0	62.3
Type of municipality															
Non-capital	66.1	65.2	67.1	5.6	5.2	6.1	23.9	23.2	24.7	80.7	80.0	81.3	60.0	59.2	60.7
Capital	63.7	62.7	64.7	5.3	4.9	5.8	23.3	22.2	24.3	80.6	79.8	81.4	62.6	61.7	63.5
Geographic region															
Less developed	69.2	68.4	69.9	4.5	4.2	4.8	20.2	19.5	20.9	83.5	83.0	84.1	55.3	54.5	56.2
More developed	63.4	62.3	64.5	6.2	5.7	6.8	25.9	25.1	26.8	79.0	78.2	79.8	63.7	62.8	64.5
Maternal education															
No formal education	71.7	69.9	73.5	6.9	5.8	8.1	24.5	22.6	26.4	85.2	83.8	86.7	54.0	51.7	56.3
Incomplete middle school	68.3	67.1	69.4	5.9	5.3	6.6	24.2	23.1	25.2	84.4	83.5	85.4	57.3	56.2	58.5
Complete middle school	66.2	64.7	67.8	5.2	4.5	6.0	23.8	22.4	25.1	80.9	79.6	82.1	61.6	60.0	63.1
Complete high school	63.6	62.3	64.9	5.5	4.9	6.1	23.7	22.6	24.8	79.0	78.1	79.9	63.0	61.8	64.1
Complete higher education	61.2	59.7	62.8	4.9	4.1	5.6	23.0	21.5	24.5	75.5	74.0	76.9	63.5	62.1	65.0
*p* trend	<0.001			0.003			0.181			<0.001			<0.001		
Total	65.6	64.8	66.3	5.6	5.2	5.9	23.8	23.2	24.4	80.7	80.2	81.2	60.6	60.0	61.2

Insufficient physical activity, alcohol consumption and regular consumption of ultra-processed foods were more frequent among girls than boys. Older adolescents presented higher frequency of three of the five risk factors evaluated, with the exception of insufficient physical activity and ultra-processed food consumption. Students whose mothers were more highly educated showed lower frequency of all risk factors (insufficient physical activity, smoking, and irregular consumption of fruits and vegetables), except regular ultra-processed food consumption. Among students attending public schools, alcohol consumption, smoking, and the irregular consumption of fruits and vegetables were more prevalent. The higher the tercile of goods and services score, the lower the prevalence of insufficient physical activity and irregular consumption of fruits and vegetables. On the other hand, we found an inverse association between the goods and services score and alcohol and ultra-processed food consumption. Adolescents who did not live with their parents were more exposed to all the risk factors assessed (Table 2).

Table 3 shows observed and expected prevalences, as well as the O/E, for all combinations of the five risk factors. Of the 32 possibilities, 13 presented an O/E above 1, which corresponded to clustering of risk factors. The combination of the five risk factors resulted in O/E of 4.17 (95% CI 3.98 to 4.37), indicating that this cluster is 4-fold higher than the expected if these behaviors were independent. The highest O/E ratio was found for the combination of smoking, alcohol and ultra-processed consumption (O/E 4.31; 95% CI 3.80 to 4.91).

**Table 3:** Clustering pattern of risk factors in Brazilian adolescents. Brazilian National Survey of School Health (PeNSE), 2015.

Number of risk factors	Presence of risk factors					Expected prevalence (%)	Observed prevalence (%)	Observed/ Expected prevalence (95% CI)
	Insufficient physical activity	Smoking	Alcohol consumption	Irregular consumption of fruits and vegetables	Regular consumption of ultra-processed foods			
5	+	+	+	+	+	0.42	1.77	4.17 (3.98, 4.37)
4	-	+	+	+	+	0.22	0.95	4.26 (4.00, 4.54)
	+	-	+	+	+	7.20	7.13	0.99 (0.97, 1.01)
	+	+	-	+	+	1.36	0.48	0.35 (0.32, 0.39)
	+	+	+	-	+	0.1	0.24	2.36 (2.08, 2.68)
	+	+	+	+	-	0.28	0.64	2.32 (2.14, 2.49)
3	-	-	+	+	+	3.78	3.90	1.03 (1.00, 1.06)
	-	+	-	+	+	0.71	0.24	0.34 (0.30, 0.38)
	-	+	+	-	+	0.05	0.23	4.31 (3.80, 4.91)
	-	+	+	+	-	0.15	0.34	2.34 (2.11, 2.60)
	+	-	-	+	+	23.07	23.43	1.02 (1.00, 1.03)
	+	-	+	-	+	1.72	1.20	0.70 (0.66, 0.74)
	+	-	+	+	-	4.68	3.16	0.67 (0.65, 0.70)
	+	+	-	-	+	0.33	0.06	0.18 (0.14, 0.23)
	+	+	-	+	-	0.89	0.21	0.24 (0.21, 0.27)
	+	+	+	-	-	0.07	0.09	1.35 (1.08, 1.64)
2	+	+	-	-	-	0.21	0.01	0.06 (0.03, 0.10)
	+	-	+	-	-	1.12	0.49	0.44 (0.40, 0.48)
	+	-	-	+	-	15.01	18.5	1.23 (1.21, 1.25)
	+	-	-	-	+	5.52	4.88	0.88 (0.86, 0.91)
	-	+	+	-	-	0.03	0.07	2.13 (1.69, 2.66)
	-	+	-	+	-	0.46	0.14	0.30 (0.25, 0.35)
	-	+	-	-	+	0.17	0.07	0.39 (0.30, 0.48)
	-	-	+	+	-	2.46	1.63	0.66 (0.63, 0.70)
	-	-	+	-	+	0.90	1.27	1.40 (1.33, 1.48)
	-	-	-	+	+	12.11	10.91	0.90 (0.88, 0.92)
1	+	-	-	-	-	3.59	3.28	0.91 (0.88, 0.94)
	-	+	-	-	-	0.11	0.02	0.20 (0.12, 0.28)
	-	-	+	-	-	0.59	0.67	1.14 (1.05, 1.23)
	-	-	-	+	-	7.88	7.24	0.92 (0.90, 0.94)
	-	-	-	-	+	2.90	3.82	1.32 (1.28, 1.36)
0	-	-	-	-	-	1.89	2.91	1.54 (1.49, 1.60)

The presence of a risk factor is shaded grey.

The combination of alcohol consumption and smoking was found in 8 of the 13 clusters identified by O>E, indicating a strong correlation between the two behaviors. The combinations: 1) insufficient physical activity, irregular consumption of fruits and vegetables, and regular consumption of ultra-processed foods (23.4%); and 2) insufficient physical activity and irregular consumption of fruits and vegetables (18.5%) were the most frequent, accounting for about half of the adolescents. However, despite its high frequency, the O/E ratio of these combinations was close to 1, and one of the combinations was not statistically significant. Only 2.9% of the adolescents did not present any risk factor, while 38.0%, 32.9%, 9.4% and 1.8% accumulated two, three, four and five, respectively. The presence of four or more risk factors was higher among girls, older adolescents, those who did not live with both parents, children of less-educated mothers, students attending public school, and residents of cities in more developed urban areas of the country (Table 4).

**Table 4:** Prevalence of co-occurrence of four or five risk factors, by socioeconomic variables. Brazilian National Survey of School Health (PeNSE), 2015.

Variables	Co-occurrence of four or five risk factors		
	%	95% CI	
Sex			
Boys	9.0	8.5	9.5
Girls	13.3	12.7	13.9
Age			
13 years or less	7.7	6.8	8.8
14–15 years	11.3	10.7	11.8
16 years or more	16.8	15.6	18.1
*p* trend	<0.001		
Skin color			
White	11.1	10.4	11.9
Non-white	11.3	10.7	11.8
Living with parents			
Both parents	9.6	9.1	10.1
One parent	13.3	12.6	14.1
Neither parent	15.3	13.8	16.9
*p* trend	<0.001		
Goods and service score			
1 tercile	10.3	9.8	10.9
2 tercile	11.9	11.2	12.7
3 tercile	11.7	10.9	12.4
*p* trend	0.002		
Type of school			
Private	9.8	8.9	10.8
Public	11.4	10.9	12.0
Area of municipality			
Rural	8.9	7.9	10.0
Urban	11.4	10.9	11.9
Type of municipality			
Non-capital	11.3	10.8	11.9
Capital	10.9	10.3	11.6
Geographic region			
Less developed	9.2	8.8	9.7
More developed	12.4	11.7	13.1
Maternal education			
No formal education	12.5	11.0	14.0
Incomplete middle school	11.8	10.9	12.7
Complete middle school	11.1	10.0	12.1
Complete high school	11.2	10.3	12.0
Complete higher education	9.9	9.0	10.9
*p* trend	0.003		
Total	11.2	10.8	11.7

## Discussion

In the present study, which included more than 100,000 Brazilian adolescents, we found that 83% of the adolescents in the study accumulated two or more NCD risk factors. The accumulation of four or more risk factors was higher in girls and in low socioeconomic groups. From the 32 possible combinations of the included five risk factors, 13 clusters were identified, indicating that these risk factors are not independently distributed in the population.

The co-occurrence of several risk factors related to NCD is not exclusive to Brazilian adolescents. In Canada, a representative sample of adolescents aged 10 to 17 years showed that 65% presented two or more risk factors, including insufficient physical activity, sedentary lifestyle, smoking, alcohol consumption and high body mass index [17]. This scenario has also been described in studies composed of adolescents in Brazilian cities and other countries [24, 45, 46].

The literature describes a large variability of analytical methods and inclusion of health behaviors in studies involving risk factor clustering [21]. Nevertheless, our findings are similar to those reported for the adult population: a strong correlation between alcohol and cigarette smoking; identification of clusters with the presence of all risk factors; and, at the other extreme, the absence of all risk factors, as well as the association of risk behaviors with social disadvantaged groups [21].

We found a higher proportion of co-occurrence of risk factors in adolescents with low socioeconomic characteristics, which should be highlighted. For instance, in Brazil, attending public school is associated with lower household income [47]. The association between risk factors and low level of maternal education may be related to income, but also reflect a lower quality of care. Maternal education has been widely accepted in the literature as an important factor in the health conditions of children [48–50]. Another feature associated with the accumulation of risk factors that may reflect low socioeconomic status and care is family structure. Single-parent families in the country are generally headed by women and tend to have lower income [47]. For this reason, the possible workload outside and within the home could adversely affect the time and quality of care offered to the adolescent.

Older adolescents were more likely to accumulate more than four risk factors. One possible explanation is the greater chance of consuming alcohol and tobacco at an older age. However, adolescents above 16 years fall outside the typical age for ninth grade, thus these students possibly have a history of grade retention and are more likely to present other characteristics of social vulnerability. Grade retention in Brazil is more frequent among boys, blacks, lower social classes, students whose parents have lower levels of education and among those whose parents do not attend school meetings nor support the accomplishment of tasks [51]. Also noteworthy is the higher prevalence of co-occurrence of risk factors in females. In Brazil, although women have higher life expectancy than men, they live longer with poorer health than men [52]. In fact, early exposure to risk factors tends to aggravate this situation, highlighting the need for NCD prevention among women.

Our study included a representative sample of adolescents enrolled in the ninth year of middle school. Brazil is a middle-income country with broad coverage for basic education. Access to education for the population aged 6 to 14 years and 15 to 19 years of age is 97.4% and 87.7%, respectively [53]. Therefore, it is plausible that our results may be applicable to all Brazilian adolescents in the studied age group. However, it is important to note that out-of-school adolescents may present a different exposure profile of the risk behaviors investigated. In addition, PeNSE included in its sample only schools with at least 15 students in the ninth grade and daytime classes. The exclusion of small schools and night classes may have led to selection bias. The impact of this bias in our results is difficult to predict.

PeNSE had a high response rate, with the exception of the variable maternal education, for which multiple imputation was performed. Although we considered only the students who answered all the original questions regarding the five risk factors, the loss was small (0.5%) and the characteristics of the sample remained similar and, therefore, generalizable for the Brazilian adolescent population. The excluded students, however, were different from the total of PeNSE, with a higher proportion of boys, adolescents over 16 years, those with mothers who did not study or did not complete elementary education, in the first tercile of goods and services score and those attending public schools.

It is important to note that this study was based on self-reported behaviors, which may have led to information bias, possibly underestimating the prevalence of risk behaviors. However, students were informed that the questionnaire was anonymous, and they answered directly from their smartphones, which may have reduced information bias. Another limitation is the dichotomization of the "risk/non-risk" behavior required for the analysis performed, which may have led to loss of information.

The clusters found in our study may indicate some forms of intervention to reduce these risk factors. The strong association between smoking and alcohol consumption among adolescents suggests that interventions related to these substances could occur simultaneously. Also, considering the age group and the low prevalence of consumption compared to adults, interventions should be mainly directed towards delaying experimentation. Although the sale and distribution of alcohol are prohibited to adolescents under 18 years in Brazil, alcoholic beverages still seem to be accessible. Thus, greater control and lower exposure to alcohol among adolescents may also affect smoking, since both substances are highly correlated.

On the other hand, the weak association identified among food related-risk factors indicates that actions in this area should cover two fronts: 1) the promotion of healthy and traditional food consumption; and 2) the avoidance of ultra-processed food consumption. Time trend data from PeNSE food consumption showed that, between 2009 and 2012, there was a decrease in the frequency of snacks and soft drinks consumption, although fruits and beans have also decreased in the same period [54].

Our results suggest possibilities of interventions related to NCD risk factors. Intervention-focused studies are needed to assess the impact of comprehensive actions on the reduction of cluster of risk factors. Even though NCD are a problem faced mainly by adults and the elderly, it is increasingly occurring at a much younger age [55, 56], impacting lower socioeconomic groups in a disproportionate manner [36]. Thus, prevention strategies should consider the first stages of life and be directed towards the population most exposed to the main risk factors.
